# A Characterization of the Oral Microbiome in Allogeneic Stem Cell Transplant Patients

**DOI:** 10.1371/journal.pone.0047628

**Published:** 2012-10-29

**Authors:** Nancy J. Ames, Pawel Sulima, Thoi Ngo, Jennifer Barb, Peter J. Munson, Bruce J. Paster, Thomas C. Hart

**Affiliations:** 1 Clinical Center, Nursing and Patient Care Services, National Institutes of Health, Bethesda, Maryland, United States of America; 2 Human Craniofacial Genetic Section, Craniofacial and Skeletal Diseases Branch, National Institute of Dental and Craniofacial Research, Bethesda, Maryland, United States of America; 3 Mathematical and Statistical Computing Laboratory, Center for Information Technology, National Institutes of Health, Bethesda, Maryland, United States of America; 4 Department of Microbiology, Forsyth Institute, Cambridge, Massachusetts, United States of America; 5 Department of Oral Medicine, Infection and Immunity, Harvard School of Dental Medicine, Boston, Massachusetts, United States of America; University of Southern California, United States of America

## Abstract

**Background:**

The mouth is a complex biological structure inhabited by diverse bacterial communities. The purpose of this study is to describe the effects of allogeneic stem cell transplantation on the oral microbiota and to examine differences among those patients who acquired respiratory complications after transplantation.

**Methodology/Principal Findings:**

All patients were consented at the National Institutes of Health, Clinical Center. Bacterial DNA was analyzed from patients' oral specimens using the Human Oral Microbe Identification Microarray. The specimens were collected from four oral sites in 45 allogeneic transplantation patients. Specimens were collected at baseline prior to transplantation, after transplantation at the nadir of the neutrophil count and after myeloid engraftment. If respiratory signs and symptoms developed, additional specimens were obtained. Patients were followed for 100 days post transplantation. Eleven patients' specimens were subjected to further statistical analysis. Many common bacterial genera, such as *Streptococcus, Veillonella, Gemella*, *Granulicatella* and *Camplyobacter* were identified as being present before and after transplantation. Five of 11 patients developed respiratory complications following transplantation and there was preliminary evidence that the oral microbiome changed in their oral specimens. Cluster analysis and principal component analysis revealed this change in the oral microbiota.

**Conclusions/Significance:**

After allogeneic transplantation, the oral bacterial community's response to a new immune system was not apparent and many of the most common core oral taxa remained unaffected. However, the oral microbiome was affected in patients who developed respiratory signs and symptoms after transplantation. The association related to the change in the oral microbiota and respiratory complications after transplantation will be validated by future studies using high throughput molecular methods.

## Introduction

The oral microbiome is one of the most complex bacterial communities within the human microbiome. Nowhere else in the human body are there so many diverse niches that support microbiota. Many different surfaces are represented in the oral cavity, including the teeth, the gingiva composed of supragingival areas and the sulcus. The epithelial surfaces of the cheek and the unique structure of the tongue are also part of this microbiome. Supragingival plaque is present on teeth and supports a distinctive bacterial community [1]. This plaque biofilm represents an entire body of research and has been described in numerous studies as not only related to major dental pathologies, caries and periodontitis but also linked to health and pulmonary infection [2]–[6]. Add to this a complex fluid, saliva, that irrigates the oral cavity and it is not surprising that the oral microbiome eludes a complete characterization. Understanding the changes that occur in this oral environment over time or because of illness or other host–related perturbations may assist scientists in discovering novel interventions to decrease disease. These novel interventions could be translated into clinical practice to improve patient care.

Allogeneic stem cell transplantation (ASCT) patients represent a population who enter the hospital for a life-saving treatment. After transplantation these patients can be neutropenic for as long as three weeks. Because of this immune compromised state and underlying disease, patients who receive an ASCT are at high risk of developing infectious complications. Infection remains one of the most serious and potentially life-threatening complications following ASCT [7], [8]. The mortality, not related to the underlying hematologic disorder but due to infections, is between 45% and 60% in the first year following ASCT [9], [10]. Recently, despite decreasing odds of developing respiratory failure after ASCT in a period from 2003–2007 (11%) as compared to an earlier period of 1993–1997 (15%), pulmonary complications remain a major cause of increased morbidity and mortality [11], [12]. This ASCT population represents a unique opportunity to examine the oral microbiome across treatment and during the first 100 days after transplantation.

We hypothesized that the oral microbiome would be changed by the transplant process. We predicted also that a relationship would exist between the oral cavity microbiota and respiratory infections. The purpose of this study was to characterize the changes in the oral microbiota of ASCT patients by comparing oral specimens collected prior to transplantation with those collected after transplantation and to compare and contrast the different oral microbial patterns of patients who develop respiratory signs and symptoms (RSS) during the first 100 days post transplantation and those who do not (NoRSS). This complex oral environment can begin to be characterized by describing the different patterns of bacterial species found in the mouths of transplantation patients using sensitive molecular methods.

## Results

Fifty patients scheduled for ASCT were consented to the study. Five patients were not eligible for transplantation due to disease progression. Of the remaining 45, eight patients developed RSS after transplantation and therefore, additional specimens were collected from these patients. The microbial profiles of oral specimens from 16 patients were analyzed using data from the Human Oral Microbe Identification Microarray (HOMIM). Of these, 11 patients' specimens were analyzed with both pre and post transplantation specimens enabling comparisons. In the 11 patients, five developed RSS post transplantation and six did not (NoRSS). Table 1 contains sampling information, sites and time points by patient. Thirty-nine specimens were obtained prior to transplantation and 74 from after transplantation. Of the 113 specimens analyzed, 61 were from patients who did not develop respiratory signs and symptoms post-transplantation and 52 specimens were from patients who developed this complication after transplantation. Four of the five patients who developed RSS were admitted to the ICU because of the severity of their symptoms. One of these patients died of transplantation-related complications. The characteristics of the 11 ASCT patients are described in Table 2. The mean age of the sample was slightly over 45-years-old (SD ±13.97), over 70% of the participants were male and most were Caucasian. The majority of the sample (82%) had a hematologic condition as an indication for transplant. One patient had breast cancer and another had HTLV-1 associated adult T cell lymphoma/leukemia. The risk of periodontal disease was assessed using the Periodontal Screening and Recording (PSR) index [13]. The risk of periodontitis was low in the sample, but gingivitis was present in all eleven patients. Two patients in the NoRSS group were assessed as being at risk for periodontitis. The mean Decayed Missing and Filled Teeth (DMFT) index, a measure of oral health, was comparable to the national average in adults [20–34 years, 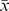 = 6.16 (SE ±0.16); 35–49 years, 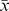 = 10.91(SE ±0.14); 50–64 years, 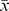 = 15.05 (SE ±0.21)] [14]. However, four patients out of the 11, all in the 50–64 year category, scored higher than the national average in adults (DMFT 23.25±6.02).

**Table 1:** Sampling information.

Study Number	Before Transplant	After Transplant			Total
	Baseline	Nadir	Engraftment	RSS	
**No Respiratory Signs and Symptoms-NoRSS**					
**11**	S, P,T	S,P, T	P,T		8
**12**	S,P,B,T	S,P,B,T	S,P,B,T		12
**13**	S,P,B,T	S,P, T	S,P,T		10
**14**	S,P,B,T	S,P,B,T	S,P		10
**15**	S,P,B,T	S,P,B,T	S,P		10
**18**	P,B,T	S,P,B,T	S,P,B,T		11
**Total NoRSS**	22	22	17		61
**Respiratory Signs and Symptoms-RSS**					
**6**	S,P, B	P,B,T	P	P,S,B,T	11
**10**	S,P,B,T	No nadir	S,P,B,T	S,P,T	
S,P,B,T					
S,P,B,T	19				
**16**	S,P,B,T	S			5
**17**	B,T	S,P,B,T		P	7
**31**	S,P,B,T		B,S,T	P,B,T	10
**Total RSS**	17	8	8	19	52
**Grand Total**	39	30	25	19	113

Sample descriptions including time and site obtained for each patient and totals. Specimens obtained from plaque (P), saliva (S), buccal brushings (B) and tongue brushings (T). Baseline specimens obtained at start of study. Nadir of absolute neutrophil count (ANC) is after transplant when ANC is at its lowest point. Engraftment is after transplant when ANC remained at 0.5×109/liter for two days. RSS represents patients that developed respiratory signs and symptoms after transplant and NoRSS represents patients who did not develop this complication.

**Table 2:** Characteristics of 11 patients.

Variables		Total patients (N = 11)	RSS (N = 5)	NoRSS (N = 6)
Gender	Male N (%)	8 (73)	2 (40)	6 (100)
Age	Mean (SD)	45.73 (±13.97)	48.60 (±5.18)	43.33(±18.80)
Ethnicity Caucasian	N (%)	5 (45.5)	3 (60)	2 (33.3)
Ethnicity AA	N (%)	3 (27.3)	1 (20)	2 (33.3)
Ethnicity Hispanic	N (%)	3 (27.3)	1 (20)	2 (33.3)
Hospital LOS	Mean (SD)	45.18 (±22.38)	56 (±20.43)	36.17 (±21.29)
ICU LOS	Mean (SD)	6.64 (±12.34)	14.60 (±15.34)	0
DMFT	Mean (SD)	12.73 (±9.49)	8.80 (±5.89)	16.00 (±11.13)
Smoking	N (%)	1 (9.1)	0	1 (16.7)
Risk of Periodontal Disease	N (%)	2 (20)*	0	2 (40)
ALC at 30 days 10^9^/Liter	Mean (SD)	1.024 (±0.569)	0.921 (±0.613)	1.109 (±0.572)
Time of neutropenia in days	Mean (SD)	8.00 (±3.60)	7.00 (±4.47)	8.83 (±2.85)
Transplant regimen (M)	N (%)	2 (18.2)	1 (.09)	1 (.09)
TBI	N (%)	2 (18.2)	1 (20.0)	1 (16.7)
Re-admit	N (%)	7 (64)	3 (60)	4 (66.7)
Mortality (100 days)	N	1	1	0

AA = African American; ALC = Absolute lymphocyte count; DMFT = Decay Missing and Filled Teeth index; NoRSS = patients who do not develop respiratory signs and symptoms; RSS = patients who develop respiratory signs and symptoms. ICU = intensive care unit; LOS = length of stay in days; Time of neutropenia is defined from nadir of white blood cell count until ANC returns to greater than 0.5×109/liter times two days. TBI = Total body irradiation the total proportion of patients (%) who received total body irradiation as part of their protocol. PSR = Periodontal Screening and Recording = risk of periodontal disease with scores range from 0–2 (2 being risk of periodontitis). The PSR is scored for 6 quadrants in the mouth with a score of Code 0–4. Scores were cut between 0 - no risk of periodontal disease; 1 - risk of gingivitis which was defined as a Code 1 or 2 in all quadrants; 2-Risk of periodontitis with Code 3 in two or more quadrants or Code 4 in at least one [46]. Re-admit = number of transplant patients who were re-admitted after their initial admission for transplant. Transplant regimen = Myeloablative (M) vs. non-myeloablative.

* Only ten patients had completed PSR scores. One patient of the 11 was neutropenic at the time of the exam.

### Description of the Oral Microbiome using the Human Oral Microbe Identification Microarray

**Figure 1:**
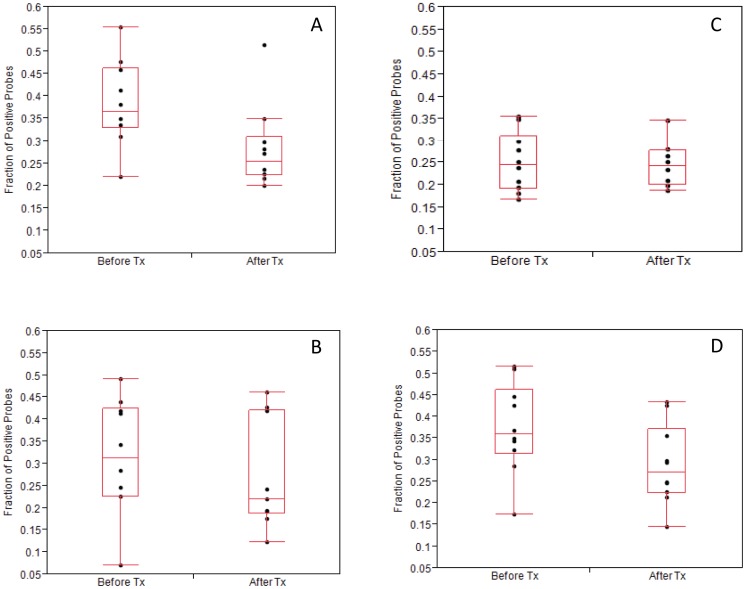
Bacterial Taxa by Site; Before and After Transplantation. Fraction of positive bacterial probes for each patient, before and after transplantation for saliva (A), plaque (B), buccal (C) and tongue (D) brushings. Each point represents a fraction of positive probes for each patient. Box plots show medians and lower and upper quartiles. The sample size varies slightly by site and by time for each site does not include all eleven patients.

Screening a total of 113 patients' specimens, HOMIM version 2 identified 155 positive probes. These positive probes are representative of eight different phyla. Four specimens were not included in the analysis as one was a tracheal aspirate that was obtained in only one patient and the other three were duplicate specimens. There were 28 specimens of saliva, 32 of plaque, 24 from buccal brushing and 29 from tongue brushings. The mean number of positive probes per patient was 22.64 (SD ±9.74) with a median of 22. The highest number of probes was in saliva (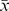 = 25.25; SD ±9.97), followed by tongue (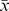 = 24.27; SD ±9.23) and plaque (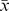 = 21.34; SD ±11.03). The number of positive probes was the lowest in the buccal specimens as compared to the other three sites (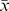 = 19. 33; SD ±7.21). There was very little difference by site in the fraction of positive probes from each patient before or after transplantation (Figure 1). Saliva was the only site that was significantly different before and after transplantation (p<.02).

**Figure 2:**
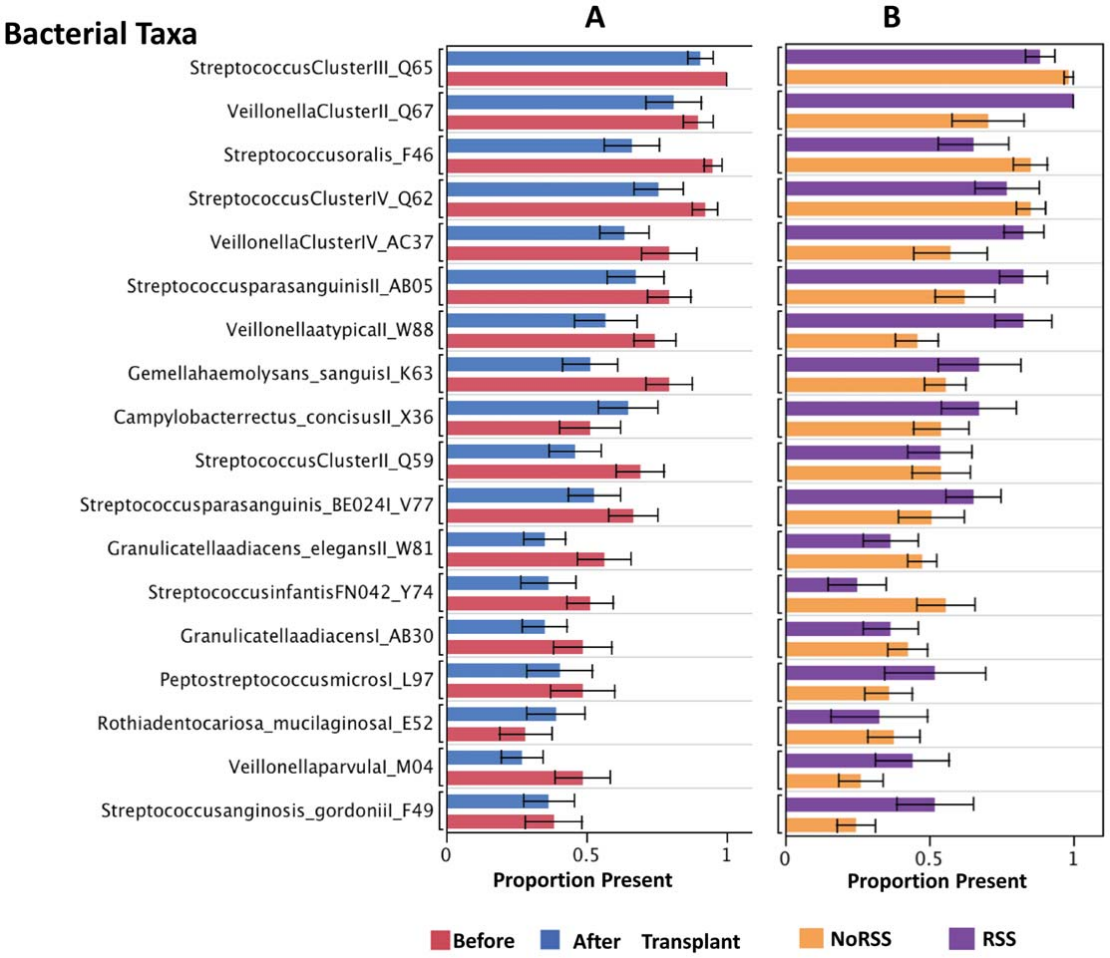
Proportion Present of Common Bacterial Taxa. Eighteen of the most prevalent bacterial probes in the entire sample and their mean proportions are shown. Panel A displays before (red) or after (blue) transplantation and panel B shows patients with NoRSS (orange) or with RSS (purple). The proportion present of each probe and standard error bars are displayed in order according to the mean proportion present (Each error bar is constructed using 1 standard error from the mean.). Each of the eighteen positive probes mean proportions were calculated for all specimens before transplant (n = 39), after transplant (n = 74) or with NoRSS (n = 61) and with RSS (n = 52). NoRSS = patients who do not develop respiratory signs and symptoms. RSS = patients who develop respiratory signs and symptoms. Streptococcus Cluster III includes all Streptococcus species; Veillonella Cluster II includes: *V.atypica, V.parvula, V.dispar*, BU083; Streptococcus Cluster IV includes: *S.anginosus, S.intermedius*, 17 bases match *S. sinensis, S.pneumoniae, S.parasanguis, S.oralis, S.mitis, S.infantis*; Veillonella Cluster IV includes: *V.parvula*, BU083, *V.dispar*; Streptococcus Cluster II includes: *S.sanguinis, S.salivarius*, strain H6.

Table 3 displays the percentage of positive probes per phyla. *Firmicutes, Proteobacteria* and *Bacteriodetes* are the three most common phyla with *Firmicutes* having the most positive probes as either of the other two phyla. These results are consistent with previous descriptions of the oral microbiome [15], [16]. A diagram or cell plot depicting the entire data set of 11 patients and their positive probes is provided (Figure S1). The specimens are identified as the rows and are ordered before and after transplant; the columns are the bacterial probes. Many of the common genera can be traced between the before and after transplant groups. The 18 most frequently occurring positive bacterial probes in specimens collected from the 11 patients is depicted in Figure 2. Panel A examines these 18 common probes before and after transplantation and Panel B compares those patients who developed RSS versus NoRSS. The majority of the organisms were from the genera *Streptococcus* and *Veillonella.* Comparing specimens collected before ASCT to those specimens collected after transplantation, sixteen of the eighteen probes had decreased presence after transplant in comparison to before transplant. Only two probes, *Campylobacter rectus* and/or *C. concisus* and *Rothia dentocariosa* and/or *R. mucilaginosa* had an increased mean presence after transplant. In contrast, ten out of 18 positive probes had an increased presence in those patients who developed RSS. All of the positive probes for *Veillonella* genera and two of the *Streptococcus* probes had increased mean presence in the RSS group. Among those probes that decreased in the RSS group were probes positive for *Streptococcus oralis* and *Streptococcus infantis*, two probes that were positive for many different species/phylotypes of *Streptococcus* (*Streptococcus* Clusters III and IV) and a probe for *Granulicatella adiacens* and/or *G. elegans*. In addition, a probe for *Rothia dentocariosa* and/or R. *mucilaginosa* had decreased presence in the RSS group samples.

**Table 3:** Percent of positive probes per phyla.

Phyla	Percent (%)	Total probes
*Firmicutes*	56.1	87
*Proteobacteria*	16.8	26
*Bacteriodetes*	14.2	22
*Actinobacteria*	7.1	11
*Fusobacteria*	3.2	5
*TM7*	1.3	2
*Spirochaetes*	0.6	1
*Synergistetes*	0.6	1
Total		155

In the analysis of 11 patients there were 155 probes divided among 8 different phyla. This chart represents the percentage of probes per phyla ordered by most common to less common.

**Figure 3:**
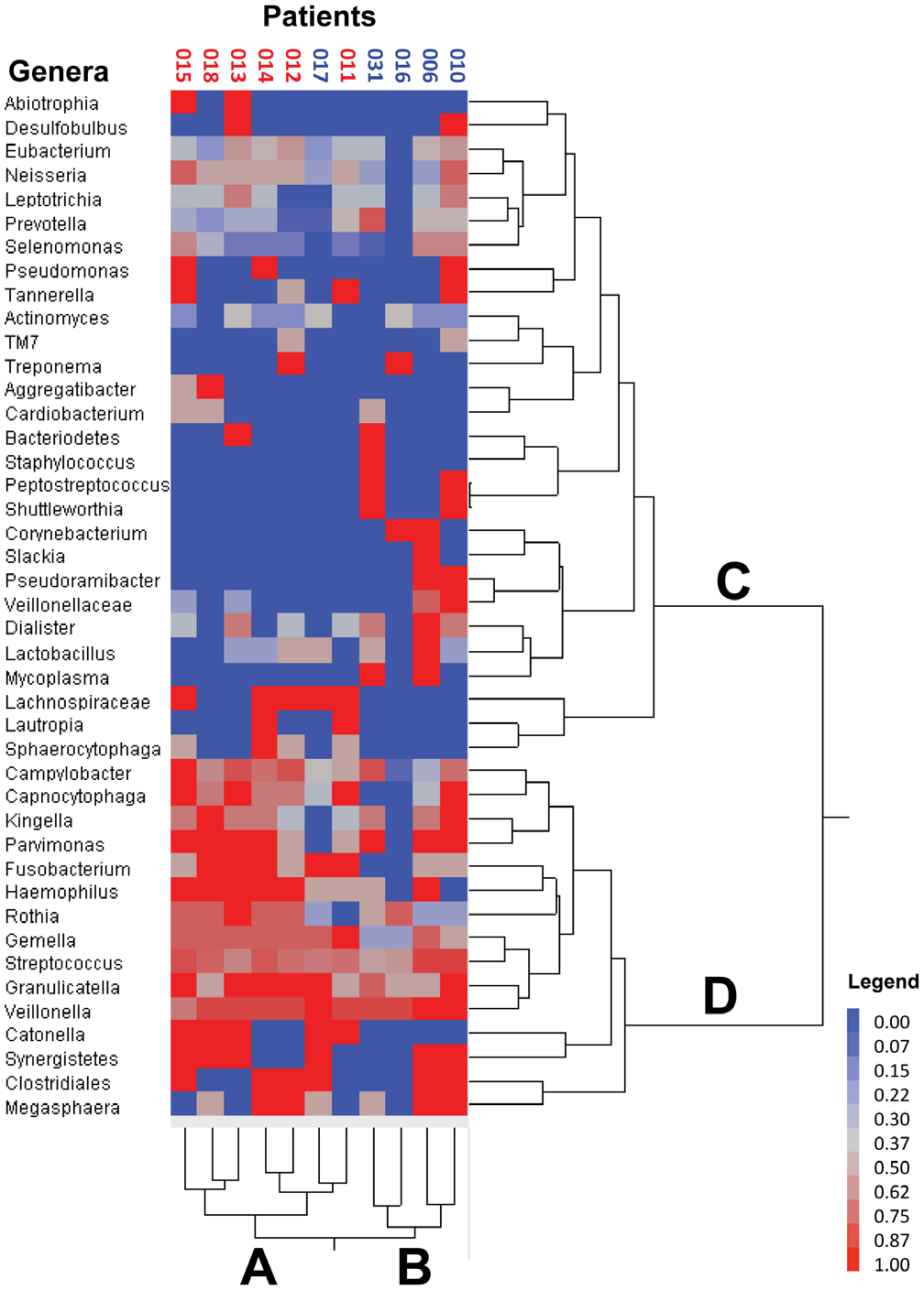
Two Way Cluster of Genera by Patient. Fraction of positive probes all sites and all times by genera and patient. The two way hierarchical cluster analysis using Ward's distance measure without standardization was calculated. Each probe was converted to a 1 (present) or a 0 (absent) based on the intensity range of the microarray data. The 155 probes were then divided among 43 genera and the proportion of positive probes within each genus was calculated. Cluster A consists of mostly NoRSS and Cluster B consists of RSS patients with one exception (017). Frequently occurring probes are in Cluster D while less frequently occurring probes are Cluster C. Supplementary material (Table S1) lists the probes and the assigned genera for this figure. NoRSS = patients who do not develop respiratory signs and symptoms. RSS = patients who develop respiratory signs and symptoms. [Note: Two probes (*Sphaerocytophaga* species strain S3) are listed in *Sphaerocytophaga* genus but are considered as synonymous with *Capnocytophaga* genera in the Human Oral Microbiome Database (HOMD) 15]. *Selenomonas* species Oral Clone CS002 oral taxon 131 remains in *Selenomonas* genus despite being re-classified into *Mitsuokella* genera Oral Clone CS002 by HOMD. *Eubacterium* PUS9170_MCE10174 remains in the *Eubacterium* genus.]

**Figure 4:**
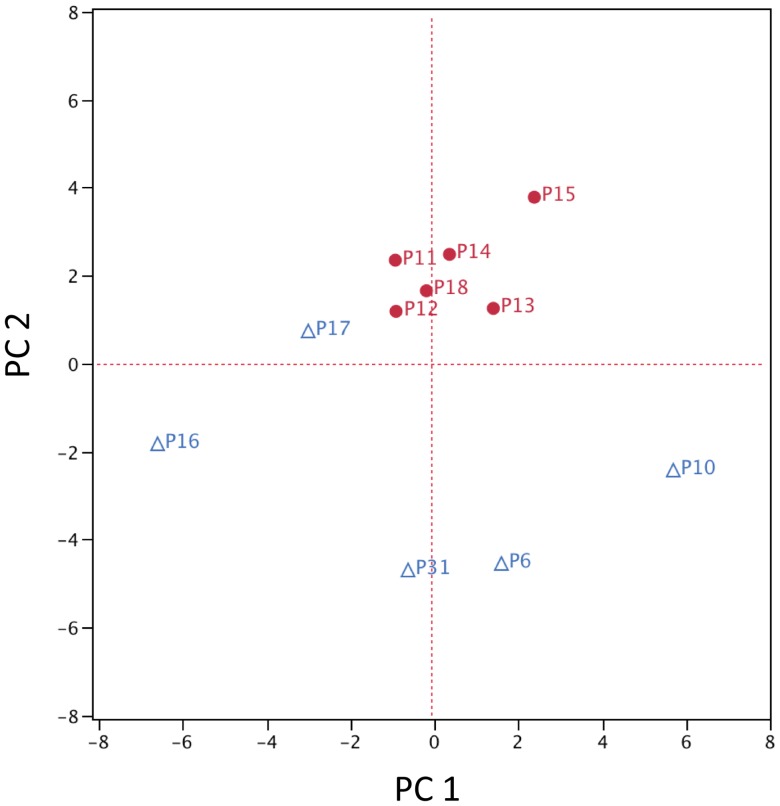
Principal Component Analysis by Genera. Patients with NoRSS are represented by red circles and those with RSS are represented with blue triangles. First principal component score (PC 1) vs. second principal component score (PC 2) plots the proportion of positive probes. Plot displays the first two principal components that represent 42.2% of the variability in the data matrix. NoRSS = patients who do not develop respiratory signs and symptoms. RSS = patients who develop respiratory signs and symptoms.

**Figure 5:**
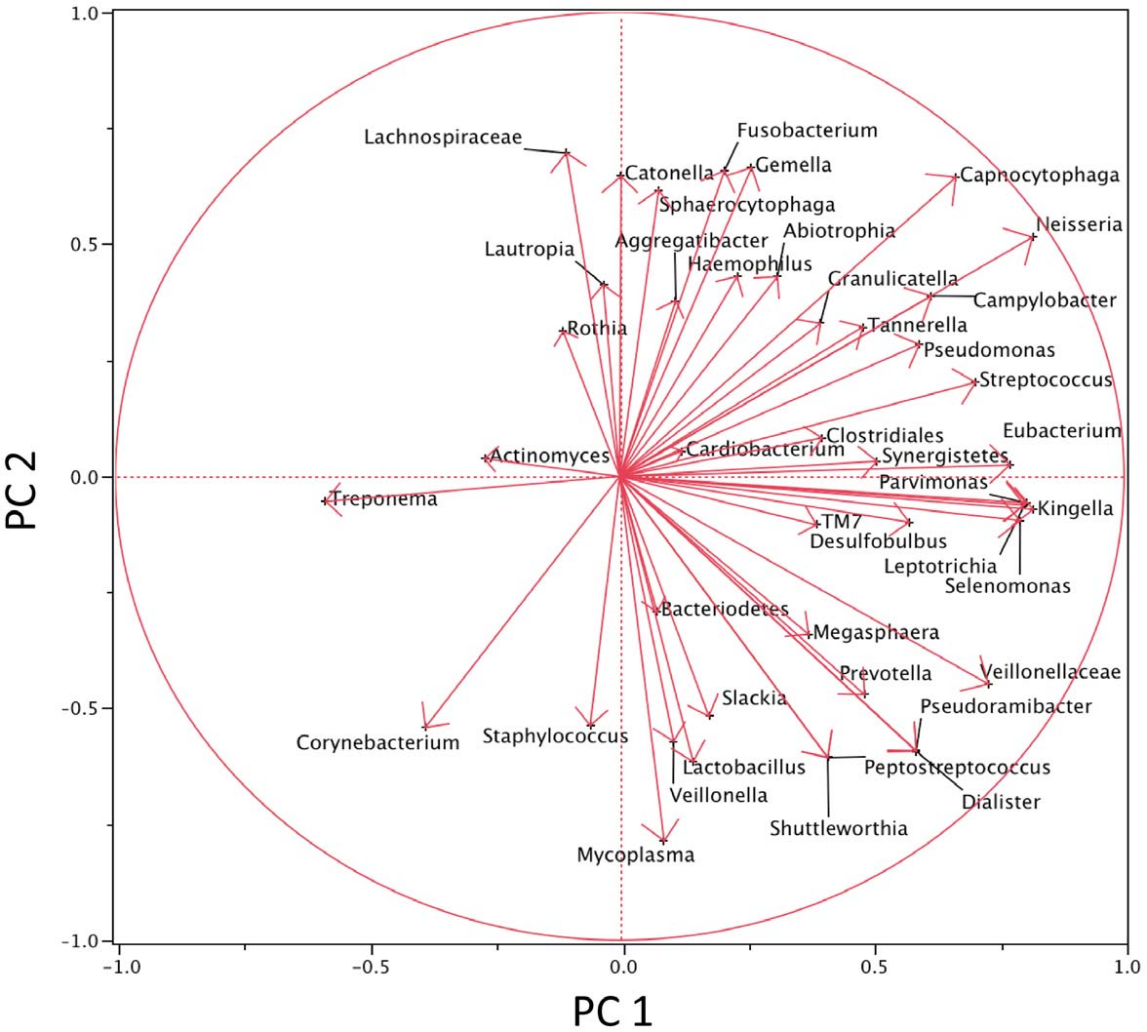
Genera Factor Loading of Principal Component Analysis. Factor loading plot of proportion of positive probes further illustrates the PCA plot from Figure 4. The loading of the 43 genera show how each genus contributed to the score plot.

There were a total of 43 genera identified by the microarray data (a list of all the probes and the included genera is in Table S1). Figure 3 is a two-way cluster, clustered by genera by patients. The sums of all positive probes by genera per patient were calculated and then an average of this number was used to determine a mean percent presence. In examining the genera, Cluster D in Figure 3 displays most of the commonly identified oral taxa, i.e. *Veillonella* and *Streptococcus,* while Cluster C displays those genera that are not as prevalent in the 11 patients, i.e. *Abiotrophia* and *Actinomyces*. Five genera, *Streptococcus, Veillonella, Granulicatella, Gemella* and *Campylobacter* were represented by positive probes in all 11 patients. Examining the clusters, by patients, Cluster B is composed of 4 of 5 patients who developed respiratory signs and symptoms after transplant. Patient 17 (P17) was the only patient who did not cluster with the other four patients who developed respiratory signs and symptoms after transplantation. The results from the two way cluster suggest a difference between the NoRSS and the RSS group. Principal component analysis (PCA) was performed using the sum of probes within each genus to confirm the difference in the oral microbiota between those patients in the NoRSS and RSS groups. Figure 4 displays the plot of the first two principal components. This plot represents 42.2% of the variability in the data set. Figure 4 displays the tight cluster of the NoRSS group while the RSS group is much more dispersed. Figure 5 displays the vectors representing the 43 genera and the positive and negative influence each contributed to the PCA plot.

**Figure 6:**
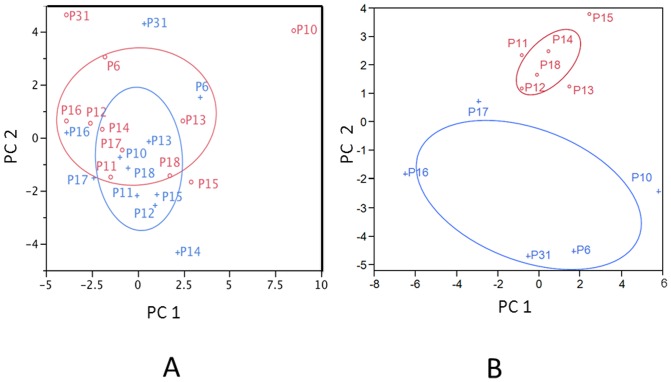
Comparisons of Principal Component Analysis Plots: Before vs. After Transplantation and NoRSS vs. RSS. Bivariate plots of principal component 1 (PC1) and principal component 2 (PC2) compare before vs. after transplantation in Plot A and RSS vs. NoRSS in Plot B. Plot A shows overlapping density ellipse between before transplantation (blue cross) and after transplantation (red open circle) groups may indicate lack of significant difference in expression between the two groups. Plot B depicts a density ellipse drawn with 50% confidence of the mean for each group. Lack of overlap of density ellipse between the RSS (blue cross) and NoRSS (red open circle) samples indicates a possible significant difference in expression between the two groups. NoRSS = patients who do not develop respiratory signs and symptoms. RSS = patients who develop respiratory signs and symptoms.

Further analysis was performed to evaluate the difference between before and after transplant. A bivariate plot of principal component one versus principal component two was calculated. A density ellipse drawn with 50% confidence of the mean for each group demonstrates overlapping ellipses consistent with a lack of difference in the two groups, that is, before and after transplant (Figure 6A). In contrast, this plot is compared to the analysis that was performed using RSS and NoRSS which shows no overlap (Figure 6B).

### Cluster Analysis

**Figure 7:**
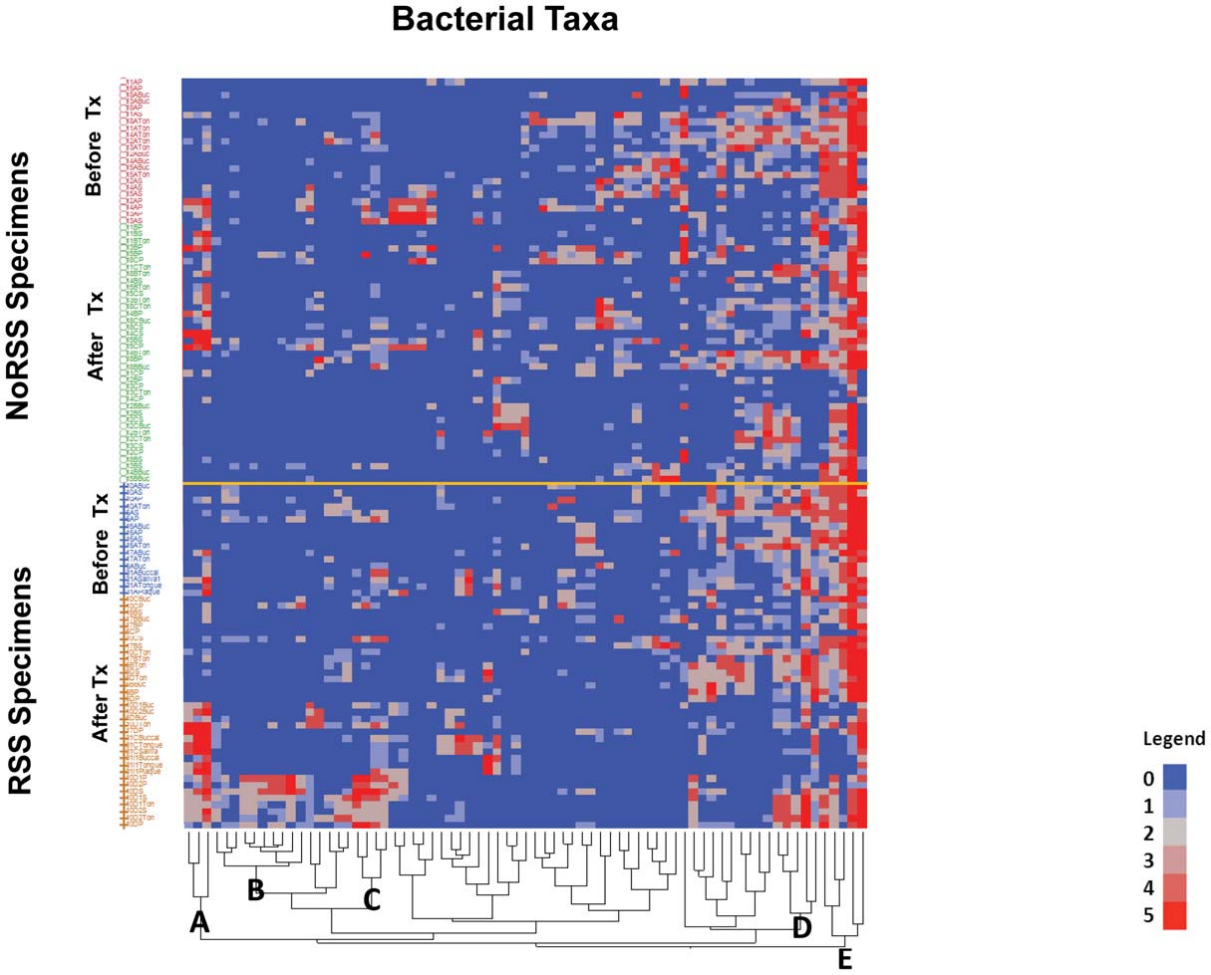
Cluster Analysis of RSS vs. NoRSS. Clinical specimens (113) from 11 patients (rows) by probes (73) (column). To be included each probe must have a median sum expression of 17 positive specimens for that probe. The sum was taken across the 113 specimens for each bacteria probe and the median was determined. A total of 73 out of 155 probes passed this requirement and were used in the two-way cluster (Table S2 lists the probes). The specimens were first divided into four groups RSS before and after transplant vs. NoRSS before and after transplant groups. Clustering was performed in each of the four groups. A second clustering was applied to probes. Cluster A specimens are composed of probes that were positive for *Camplylobacter rectus* and/or *C. concisus*. Cluster B is composed of positive probes *Campylobacter rectus* and/or *C. concisus,* two probes for *Streptococcus,* namely *Streptococcus intermedius* and/or *S. constellatus* and *Streptococcus anginosus* and/or *S.intermedius*. Also included in Cluster B are positive probes for *Dialster invisus, Eubacterium infirmum* and five different probes for the genus *Selenomonas*. Cluster C is composed of positive probes for *Parvimonas micros Dialaster invisus* and *Eubacterium saburreum*. Cluster D and E represent common oral bacterial taxa. Analysis was performed using JMP (Cary, NC), hierarchical two-way clustering with Ward's distance measure applied to the normalized fluorescence intensity signal from the microarray data, on a 0 to 5 scale. NoRSS = patients who do not develop respiratory signs and symptoms. RSS = patients who develop respiratory signs and symptoms.

We compared the bacterial probe cluster analysis between the six patients in the NoRSS group to the five patients in the RSS group (Figure 7). We selected 73 probes that included many of the frequently occurring bacterial taxa (a list of probes that are included in this analysis are in Table S2). In order to filter out probes with very little presence, a median sum expression of 17 or more positive specimens per probe was taken. Seventy-three probes met this criterion. All 113 specimens were placed in the rows according to the group in which they belonged, namely RSS or NoRSS and before or after transplantation. An orange line separates the two groups, RSS and NoRSS. Some distinct differences can be identified between the RSS and NoRSS groups. Cluster A in the after transplant RSS section is a block of specimens composed of many of the probes that were positive for *Campylobacter rectus* and/or *C. concisus*. These bacteria taxa are not present in many of the NoRSS after transplant group. Cluster B is composed of positive probes for *Campylobacter rectus* and/or *C. concisus,* two probes for *Streptococcus,* namely *Streptococcus intermedius* and/or *S. constellatus* and *Streptococcus anginosus* and/or *S.intermedius*. Also included in Cluster B are positive probes for *Dialster invisus, Eubacterium infirmum* and five different probes for the genus *Selenomonas*. These organisms are not present in most of the NoRSS specimens. Cluster C in the RSS after transplant group represents another unique group of positive probes. Cluster C is composed of positive probes for *Parvimonas micra*, *Eubacterium saburreum* and *Dialister invisus*. In the NoRSS group, in many patients, these same probes are negative. In contrast, to the differences observed between the RSS and NoRSS groups, Cluster D and E contain many of the most frequently occurring probes such as *Streptococcus*, *Veillonella* and *Gemella* species. These organisms did not change based on group designation or time point and are present consistently in the hierarchical cluster. Generally, these common bacterial taxa can be traced from the top to the end of the cluster, including both the RSS and NoRSS groups.

## Discussion

This descriptive study is the first to investigate the longitudinal effects of ASCT on the oral microbiome. Compared to baseline specimens collected prior to transplantation, the common core bacteria profiles in patients' oral cavities changed very little after transplantation. Following stem cell transplantation, the greatest change occurred in the oral microbiome of patients who developed respiratory signs and symptoms.

This study lends support to the concept of a core oral microbiome that has been proposed and supported by a number of studies [16], [17]. The presence of common oral organism such as *Streptococcus*, *Gemella* and *Veillonella* before and after transplant and in the majority of specimens supports this concept. In both of the cluster analyses, (Figure 3 Cluster D and Figure 7 Cluster D, E) an area that represents these common oral bacteria remains relatively stable. In the bacterial taxa identified in this analysis (Figure 2), all common species are identified in specimens that were obtained before and after transplant. In addition, when subjected to principal component analysis, Figure 6A supports this assertion in that the two groups, before and after transplant are not different. This is evidenced by the overlap in the density ellipses. Examining Figure 6A, most of the patients' before transplantation (blue crosses) and after (red circles) specimens are in close proximity to each other in this plot.

In other studies examining the oral microbiome, the core microbiome is composed of identical organisms across unrelated, healthy individuals [17], [18]. In one study, over 500 different species-level phylotypes were identified. Despite this diversity, 72% of the genus level or greater was shared among three healthy individuals, leading the authors to conclude that there was a core oral microbiome [17]. If a core oral microbiome exists and can be defined, then changes over time and between individuals may precede disease states.

Preparations for stem cell transplantation involve conditioning chemotherapy and, in some, total body irradiation. Prophylactic and treatment antibiotics were administered to the majority of patients. Post transplantation, all the patients experienced a significant period of neutropenia (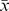 = 8.00; SD ±3.60 days) and lymphocytopenia (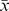 = 1.024; SD ±0.569 absolute lymphocyte count at 30 days) (Table 2). The final outcome of stem cell transplantation is that the recipient receives the donor's immune system. Remarkably, despite these intense interventions and a new immune system, the oral bacterial microbiome remained fairly stable. As discussed, the 18 most common probes remain present before and after transplant, however, the mean presence of sixteen of the eighteen probes decreased after transplant (Figure 2A).

Our data analysis indicates a more pronounced difference between the oral taxa identified in the RSS and NoRSS groups. This finding is demonstrated in the principal component analysis in Figure 4 with its tight grouping of the NoRSS group as compared to the dispersion of the RSS group and in the bivariate plot and the lack of overlap in Figure 6B. In this plot, the majority of the NoRSS group forms a tight cluster in the small ellipse in contrast to the wide dispersion of the specimens from the RSS group. This further supports the assertion that the bacterial taxa in the patients who developed respiratory complications after transplantation were to some extent different than in patients who did not develop this complication.

We used cluster analysis to divide data into meaningful and useful groups so that patterns could be identified. This technique has been used to analyze differences in other complex microbial communities [19]–[21]. Examining Figure 7, a different pattern of positive probes can be seen in Cluster A, B and C when comparing NoRSS and RSS groups. In this study, bacterial taxa were grouped into clusters and patterns emerged that were not apparent when examining individual results. Results of cluster analysis provided evidence that the bacterial patterns in patients who developed respiratory signs and symptoms after transplantation were distinct from baseline or from those patients who did not develop this complication. The five patients who developed respiratory complications after transplantation had a different pattern of positive probes. This is especially true in the specimens obtained from the RSS group after transplantation. Development of respiratory signs and symptoms after transplantation, not the transplant process itself, appears to be associated with an identified difference in the oral microbiota.

Many of the common oral species represented by two of the *Streptococcus* Clusters (III and IV) and *Streptococcus oralis* decreased in patients who developed RSS (Figure 2B). As in the before and after transplant groups, this change could have been caused by the effects of antibiotics and the accompanying increase in other bacteria because ten out of the 18 other common probes increased in those patients who developed respiratory complications (Figure 2B). Other studies which examine complex microbial environments have documented temporal changes related to treatment with antimicrobial therapies [22], [23]. Antibiotic therapy prevents the growth of some organisms but also changes the microbial environment in complex and yet unknown ways [24].

In the two way cluster by patients and genera (Figure 3), Cluster B is composed of four of five patients who developed respiratory signs and symptoms after transplantation. P17 is the only RSS patient not included in this cluster. In the PCA plot (Figure 4) P17 (blue triangle) appears at the margin of the tightly plotted NoRSS group, despite this patient belonging to the RSS group. The bacterial taxa identified in this patient were more similar to the NoRSS transplant patients than the RSS group. One explanation for this finding is that for this patient (P17), the majority of specimens analyzed were obtained prior to development of respiratory signs and symptoms.

Figure 5 plots the vectors representing the 43 genera. Genera represented by vectors in the right upper quadrant were major contributors to the weight of the NoRSS group in Figure 4. The majority of the common bacterial taxa were located in the right upper quadrant with only a few genera identified in the right lower quadrant (*Veillonella,* one of the most common genera, and *Lactobacillus*). In contrast, some taxa such as *Bacteriodetes* and *Shuttleworthia* might have contributed to the diversity in the RSS group and its unique appearance in the PCA plot.

Changing patterns of the oral microbiome have been documented in other high risk patients with pulmonary infections such as the critically ill [25]–[30]. In ASCT patients, a recent study which examined the gut microbiota in patients after transplantation found severe disruption in this environment potentially related to inflammation [31]. We did not document any severe disruption of the microbiota related solely to transplantation.

In the current study, the use of microarray as the discovery method limits the ability to identify unique bacterial taxa that may be present in this microbiome. Because the HOMIM microarray is constructed with multiple probes identifying key oral bacterial species and phylotypes, other potential limitations and biases may exist. Approximately 30% of the targeted species have a second probe located on the microarray [32]. However, this duplication of probes would have affected all of the groups equally. A recent study comparing the HOMIM microarray to 454 pyrosequencing found the HOMIM microarray was accurate in identifying commonly detected oral taxa at the genus level [33]. Correlations between the two methods were high (0.70–0.84) when comparing common genera. Additionally, the study by Ahn and others reported that 37 genera detected by both methods represented 98% of the classified bacteria [33].

In summary, two key findings have been identified. First, although differences were noted, the changes in the most common probes used in the HOMIM microarray demonstrate consistency before and after transplant. The oral microbiome appears to have been changed minimally by the transplantation process. Second, development of respiratory complications after transplantation appears to be associated with changes in the oral microbiome. Future studies using high throughput molecular methods could validate these results and confirm the stability of the oral microbiome over time and the changes associated with respiratory complications post-transplantation. This complex oral environment requires further studies focusing on these and other factors that influence or change this community of oral organisms.

## Materials and Methods

### Subject Population

The study was conducted at the National Institutes of Health, Clinical Center in Bethesda, Maryland. Patients scheduled to obtain an ASCT between July 9, 2007 and May 22, 2008 were asked to participate in this study (Protocol 07-CC-0153). All patients who met the inclusion criteria (18 years of age or older and scheduled for transplantation) and possessed none of the exclusionary criteria (recent oral or facial trauma, sickle cell or chronic granulomatous disease) were asked to participate in the study. Four eligible patients who met the inclusion criteria chose not to participate. All patients in this study were enrolled also in experimental transplant protocols that directed their care and treatment. Permission to conduct this study was obtained through the intramural Institutional Review Boards of the National Institute of Dental and Craniofacial Research (NIDCR) and the University of Maryland, Baltimore. Written informed consent was obtained prior to collection of specimens and data. For the majority of patients, consent was obtained in the dental clinic when patients arrived for screening prior to transplantation. Clinical data were collected from patients' charts, the electronic medical record (Clinical Research Information System) and physical assessment.

Initial demographic information and baseline oral samples were collected prior to admission to the hospital for the transplantation. These baseline specimens served as the patients' own controls. Specimens of saliva, supragingival plaque and mucosal brushings from the tongue and buccal surfaces were collected. Figure S2 is the timeline of the sampling process. In addition to baseline specimens, all patients had specimens collected at two scheduled times after transplantation; at the nadir of the patients' absolute neutrophil count; and after myeloid engraftment when the absolute neutrophil count was greater than 0.5×10^9^/liter for two days.

If the patient developed any respiratory signs and symptoms (Table S3) with an inpatient or ICU admission, additional specimens from all sites were collected within 24 hours (h) and were repeated twice every 48 h. If a patient was admitted to the ICU and endotracheal intubation occurred, additional specimens were collected from all oral sites including tracheal aspirates. Again, these specimens were collected within 24 h of intubation and every 48 h for two collections. In addition to the specimens, data collection included reason for admission, proposed etiology of respiratory signs and symptoms and collection of the Acute Physiologic and Chronic Health Evaluation (APACHE) [34] score, which is a measure of critical care acuity.

The primary investigator (NA) performed all data and specimen collection except for the Decayed Missing and Filled Teeth (DMFT) index [35], and the Periodontal Screening and Recording (PSR) [13], which were obtained by dentists as part of the dental screening exam for transplantation. The PSR was not performed if the patient was neutropenic. Other measures were collected at baseline and with every specimen collection time included: the modified Beck Oral Assessment Scale (BOAS) [36]; the Mucosal Plaque Score (MPS) [37]; the World Health Organization Oral Mucositis Scale [38]; and the Graft versus Host Disease scale [39]. These data are not reported here.

### Specimen Collection

Approximately one ml of saliva was collected in sterile microcentrifuge tubes. Saliva was sampled using a small, suction collection device composed of disposable sterile tubing and a reservoir [40]. The reservoir was cleaned and gas-sterilized after each collection. Supragingival plaque was collected with a sterile plastic applicator from posterior interproximal sites, if accessible. Plaque, buccal and tongue brushings were placed in sterile microfuge tubes with 150 µl of TE buffer [41] composed of 10 millimoles of TRIS with 1 milllimole of EDTA. Two other sites in the oral cavity, buccal mucosa and dorsum of the tongue, were sampled using a sterile buccal brush (Cytopak®, Medical Packaging Corporation). The buccal brush was stroked for at least 5–10 seconds over the mucosal tissue inside the oral cavity against the surface of the cheek. Both cheeks were sampled if there were no contraindications to this, such as oral lesions. The dorsum of the tongue was sampled in a similar manner by selecting a 1 cm^2^ area at the center of the tongue [42]. The surface was gently brushed for 5–10 seconds and the sample was placed in the microfuge tube with TE buffer. Oral specimens were collected at least two hours after eating or any oral care. Sterile scissors were used to cut off the sampling stick and the brush was sealed in the microfuge tube. Prior to placing the brush specimens of the tongue and buccal mucosa in the freezer, the tubes were vortexed for 30 seconds. Specimens immediately were placed on ice and transported to storage at −80°C.

### DNA Extraction and Purification

Bacterial DNA was isolated and prepared using the manufacturer's instruction for Epicentre MasterPure Kit for DNA extraction (Epicentre Biotechnologies, Madison, Wisconsin). DNA products were examined for concentration and purity using the NanoDrop 1000 (Nanodrop Products, Wilmington, Delaware). The universal 16S rRNA primers [RW0 (Forward) AACTGGAGGAAGGTGGGGAT and DG74 (Reverse) AGGAGGTGATCCAACCGCA] were used to test bacterial DNA quality before HOMIM analysis at Forsyth Institute, Boston.

### Human Oral Microbe Identification Microarray

The HOMIM are glass slides spotted with unique nucleotide sequences that serve as probes for specific organisms [43]. The microarray uses reverse-capture hybridization of fluorescently-labeled 16S rRNA products. A nested PCR reaction is used to incorporate the labeled nucleotide (Cy3-dCTP), the fluorescent material, into the isolated DNA from the clinical samples, prior to hybridization. The probes were 18 to 20 bases in length that represent unique bacteria and over 30% of the targeted species have a second probe. The 16S rRNA oligonucleotide reverse capture probes were printed on 25×76 mm aldhyde-coated glass slides using the OmniGrid Arrayer (GeneMachines, San Carlos, CA). Version 2 (lot HOMIM v09.07) was used in this analysis and can identify 257 unique bacterial species using over 400 probes. Additional information concerning HOMIM is available at (http://mim.forsyth.org/homim.html). A list of all the bacterial taxa and clusters used in HOMIM Version 2 is available in Supporting Information (Tables S4). Using the fluorescently-labeled 16S rRNA products, the results were translated to a bar code format and normalized by comparing individual signal intensities to the average of signals from universal probes. The bands colors correspond to presence or absence and band intensities were scored from zero to five. The microarray data have been deposited in NCBI's Gene Expression Omnibus [44] and are accessible through GEO Series accession number GSE34439 (http://www.ncbi.nlm.nih.gov/geo/query/acc.cgi?acc=GSE34439).

### Statistical Analysis

Descriptive and univariate statistics were used to compare demographic data and the output from the HOMIM. The MSCL Analyst's Toolbox developed by two of the authors (JB, PJM) in the JMP, Statistical Discovery Software (SAS Institute Cary, NC, 2008) was used for all the analyses. The average of positive bacterial probes, before and after transplantation were calculated for each of the four oral sites namely saliva, plaque, buccal and tongue brushings. The sites had equally dispersed number of specimens among them (28 specimens of saliva, 32 of plaque, 24 from buccal brushing and 29 from tongue brushings). The mean proportion of the 18 most common probes were calculated from all of the 113 specimens collected over all probes, sites and time points. These semi-quantitative values (0–5), based on the intensity of the fluorescence from the microarray data, were re-coded from the original data to a 0 if 0 and a 1 if 1 to 5. For each probe, the average number of positive probes for each patient before and after transplant was obtained. The average over patient was calculated for each of the 18 highly present probes and weighted to account for the number of specimens. A similar procedure was used for the mean proportion of RSS and NoRSS.

The two way cluster analysis (Figure 3) was performed using Ward's distance measure without standardization [45]. Each probe was re-coded as above (0 if 0 = not present; 1 if 1–5 = present). The 155 probes grouped by genus and the proportion present per patient were calculated by computing the sum over all probes within genus and then dividing by the total number of probes within that genus. Each small square in the heat map represents each genus per patient over the total probes in each patient. This is the percentage of presence. This summarized data was also used in the PCA (Figure 4). The vectors of the 43 genera were obtained (Figure 5). Hierarchical cluster analysis using Ward's distance measure without standardization was performed on patients with and without respiratory symptoms (Figure 7). The rows of specimens were categorized as before and after transplant as well as NoRSS and RSS groups. In the original dataset of 16 patients, if an organism was present in only one specimen, that probe was excluded from the analysis.

## Supporting Information

Figure S1 **Cell Plot.** This cell plot represents the entire data set. The specimens are ordered before and after transplant and are the rows. The major genera are the columns.
(TIF)

Figure S2 **Timeline of Specimen Collection.** The schema of the study highlights timing of specimen collection. The red triangle in the figure denotes the complication that prompted additional specimen collection. Specimens of saliva, dental plaque and mucosal brushings were collected at three time intervals: 1) before hospitalization; 2) after stem cell infusion, within 48 hours at the nadir of their absolute neutrophil count and 3) after myeloid engraftment when the absolute neutrophil count is greater than 500 (0.5×10^9^/liter) for two days. If the patient developed respiratory signs and symptoms with an inpatient or intensive care unit admission, additional specimens were collected.
(TIF)

Table S1 **Probes and Genus Designation.** This spreadsheet lists all the positive probes identified in the data set and the corresponding genus.
(XLS)

Table S2 **Bacterial Probes in** **Figure 7** **.** The bacterial probes that were selected for Figure 7 are listed. These probes were used to develop the hierarchical cluster analysis.
(DOCX)

Table S3 **Respiratory Signs and Symptoms.** Respiratory signs and symptoms used as the indicator for additional specimen collections and for classification of respiratory complications after transplantation.
(DOCX)

Table S4 **Bacterial Probes and Clusters in HOMIM Version 2.** All bacterial probes and clusters (groups of bacteria, combined in one probe) that were included in the Human Microbe Identification Microarray (HOMIM) are identified.
(DOC)
