# Supplementary figures and images for: A Characterization of the Oral Microbiome in Allogeneic Stem Cell Transplant Patients
Source: PLoS One. 2012 Oct 29;7(10):e47628. doi: 10.1371/journal.pone.0047628 (PMC3483166; doi:10.1371/journal.pone.0047628)

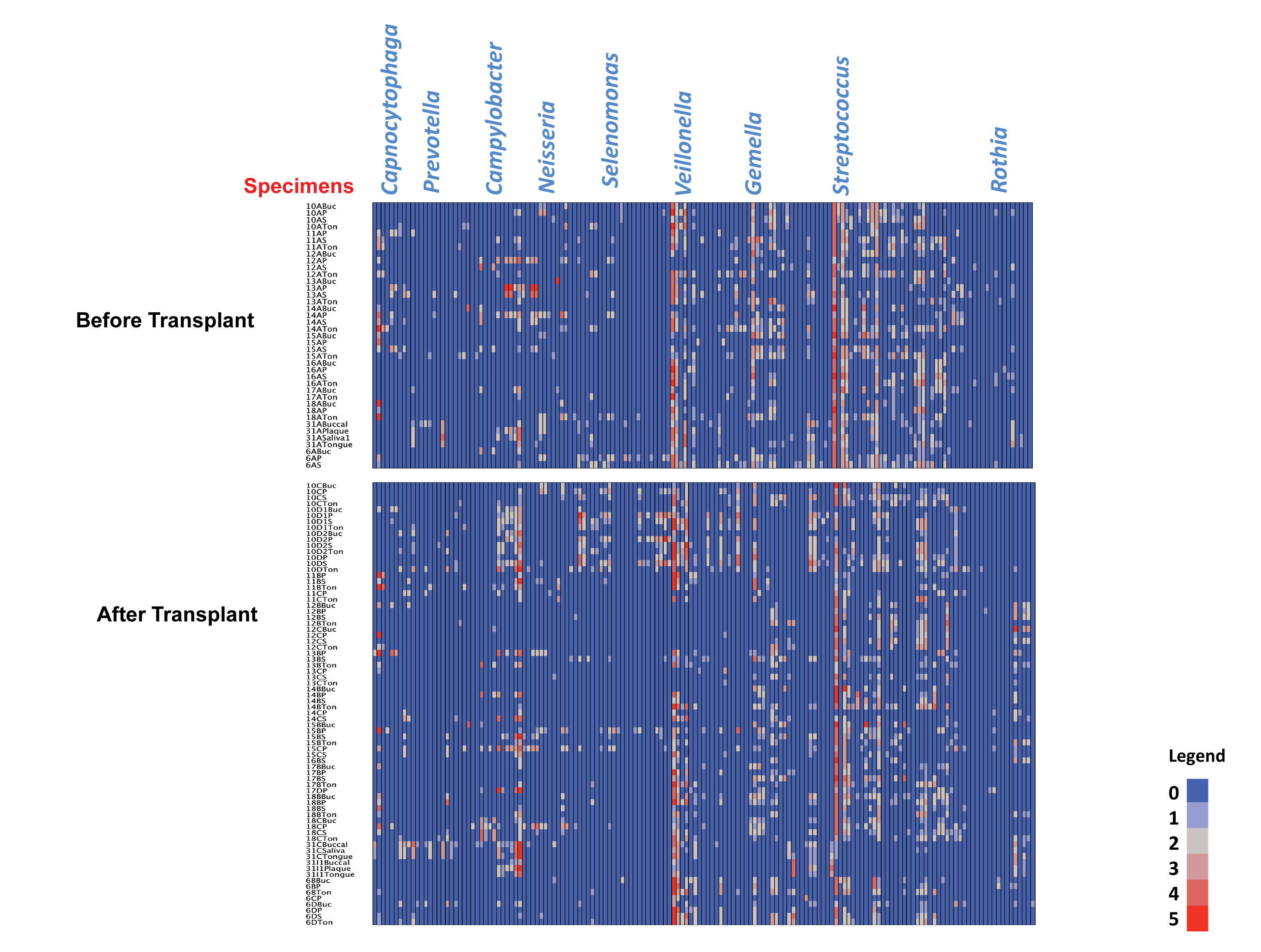

Supplement: Figure S1 — Cell Plot. This cell plot represents the entire data set. The specimens are ordered before and after transplant and are the rows. The major genera are the columns. (TIF) [file pone.0047628.s001.tif]
