# Supplementary material for: A Characterization of the Oral Microbiome in Allogeneic Stem Cell Transplant Patients
Source: PLoS One. 2012 Oct 29;7(10):e47628. doi: 10.1371/journal.pone.0047628 (PMC3483166; doi:10.1371/journal.pone.0047628)
Supplement: Table S2 — Bacterial Probes in Figure 7 . The bacterial probes that were selected for Figure 7 are listed. These probes were used to develop the hierarchical cluster analysis. (DOCX) [file pone.0047628.s004.docx]

**Table S2**

CampylobacterconcisusI_046

Campylobacterrectus_concisusI_T86

Campylobacterrectus_concisusII_X36

CampylobacterconcisusII_X33

Streptococcusanginosus_intermediusII_AB82

Streptococcusintermedius_constellatusI_F48

DialisterinvisusII_AA94

EubacteriuminfirmumI_Y45

SelenomonasspEW084_DS071I_Q52

SelenomonassputigenaI_K65

SelenomonassputigenaII_AB04

SelenomonasstrainGAA14I_AB58

SelenomonasinfelixI_O54

FusobacteriumnucleatumsspolymorphumI_P89

PrevotellaspDO039I_Q94

MegasphaeramicronuciformisII_AA57

Streptococcusparasanguinis_BE024II_R17

Veillonellaatypical_W04

DialisterinvisusI_P73

EubacteriumsaburreumI_W21

PeptostreptococcusmicrosI_L97

PeptostreptococcusmicrosII_V05

CampylobactergracilisI_Q04

CampylobactergracilisII_X34

CampylobactershowaeI_W36

CampylobactershowaeII_X35

Capnocytophagasputigenal_W46

LactobacillusClusterI_W94

PrevotellaClusterVI_AA44

Prevotellamelaninogenica_BE073I_T81

PrevotellaspBE073_FO012_AA53

PrevotellaClusterI_Y64

LactobacilliusgasseriI_V86

Rothiadentocariosa_mucilaginosal_E52

RothiamucilagionosaII_AB63

RothiamucilaginosaIII_AB62

Streptococcusaustralis_DialisterinvisusI_U50

Eubacteriumyurii_A3MTI_W84

NeisseriaelongataI_AA75

NeisseriaflavescensI_AA76

KingellaoralisI_O86

KingellaoralisII_AA77

StreptococcussanguinisI_AB75

StreptococcussanguinisII_AB78

Haemophilusparainfluenzae_aphrophilusI_W79

HaemophilusspBJ095I_AA97

GemellamorbillorumI_K64

GemellamorbillorumII_AB09

StreptococcisClusterI_AB98

GranulicatellaelegansI_AB28

GranulicatellaelegansII_AB29

Streptococcuscristatus_infantisI_X08

Streptococcusmitisbiovar2I_Q64

CapnocytophagaClusterII_AA89

Eubacteriumsaburreum_BE088I_AB50

Veillonellaparvulal_M04

Streptococcusanginosis_gordoniiI_F49

StreptococcusClusterII_Q59

Streptococcussalivarium_FO042I_E34

Granulicatellaadiacens_elegansII_W81

GranulicatellaadiacensI_AB30

StreptococcusaustralisT1E5II_AB83

StreptococcusinfantisFN042_Y74

Streptococcusparasanguinis_BE024I_V77

StreptococcusparasanguinisII_AB05

Streptococcusparasanguinis_sinensisIII_AB99

VeillonellaatypticaII_W88

VeillonellaClusterIV_AC37

Gemellahaemolysans_sanguisI_K63

StreptococcusClusterIV_Q62

Streptococcusoralis_F46

StreptococcusClusterIII_Q65

VeillonellaClusterII_Q67
