# Supplementary material for: A Characterization of the Oral Microbiome in Allogeneic Stem Cell Transplant Patients
Source: PLoS One. 2012 Oct 29;7(10):e47628. doi: 10.1371/journal.pone.0047628 (PMC3483166; doi:10.1371/journal.pone.0047628)
Supplement: Table S3 — Respiratory Signs and Symptoms. Respiratory signs and symptoms used as the indicator for additional specimen collections and for classification of respiratory complications after transplantation. (DOCX) [file pone.0047628.s005.docx]

**Table S3 Respiratory Signs and Symptoms**

| Respiratory Signs & Symptoms | Definition |
| --- | --- |
| **Tachypnea** | **Increase in rate by 10% over baseline** |
| **Dyspnea**  **Shortness of Breath**  **Increased sputum production** | **Patient complains of difficulty breathing and /or shortness of breath**  **Productive cough** |
| **Fever** | **Fever of 38 degrees or greater** |
| **Hypoxemia** | **Decrease of at least 10 % in pulse oximetry value** |
| **Pulmonary infiltrates on x-rays that were not present previously** |  |
| **Use of supplemental O2** | **Patient had not required supplemental oxygen previously** |
