# Supplementary material for: A Characterization of the Oral Microbiome in Allogeneic Stem Cell Transplant Patients
Source: PLoS One. 2012 Oct 29;7(10):e47628. doi: 10.1371/journal.pone.0047628 (PMC3483166; doi:10.1371/journal.pone.0047628)
Supplement: Table S4 — Bacterial Probes and Clusters in HOMIM Version 2. All bacterial probes and clusters (groups of bacteria, combined in one probe) that were included in the Human Microbe Identification Microarray (HOMIM) are identified. (DOC) [file pone.0047628.s006.doc]

**Table S4 Version II HOMIM All Probes and Clusters**

| Actinobacillus actinomycetemcomitans |
| --- |
| Actinobaculum sp. EL030 |
| Actinomyces georgiae |
| Actinomyces gerensceriae |
| Actinomyces israelii |
| Actinomyces naeslundii I ATCC 12104 |
| Actinomyces naeslundii II |
| Actinomyces odontolyticus |
| Actinomyces sp. AP064 |
| Actinomyces sp. EP005 |
| Actinomyces sp. EP011 |
| Actinomyces sp. EP053 |
| Corynebacterium durum |
| Actinomyces sp. strain B19SC |
| Actinomyces sp. strain B27SC |
| All Bifidobacterium (Genus-specific) |
| All Streptococcus (Genus-specific) |
| All Synergistes (Genus-specific) |
| All Treponema(Genus specific) |
| All Veillonella (Genus-specific) |
| Atopobium parvulum |
| Atopobium rimae |
| Atopobium sp. C019 |
| Bacteroidetes sp. _X083 |
| Bacteroidetes sp. AU126 |
| Bifidobacterium dentium |
| Bifidobacterium sp. CX010 |
| Bifidobacterium sp. strain A32ED |
| Brevundimonas diminuta |
| Bulledia extructa |
| Solobacterium moorei |
| Campylobacter rectus |
| Campylobacter showae |
| Campylobacter curvus |
| Campylobacter concisus |
| Campylobacter gracilis |
| Campylobacter concisus |
| Capnocytophaga sp. BM058 |
| Capnocytophaga sp.BU084 |
| Capnocytophaga sp.DZ074 |
| Capnocytophaga sp.DS022 |
| Capnocytophaga sp. BU084 |
| Capnocytophaga granulosa |
| Capnocytophaga gingivalis |
| Capnocytophaga sp. _X066 |
| Capnocytophaga sp. _X089 |
| Capnocytophaga sp. AA032 |
| Capnocytophaga sp. BB167 |
| Capnocytophaga sp. ochracea |
| Capnocytophaga sp. BR085 |
| Capnocytophaga sp. DS022 |
| Capnocytophaga sp. S3 |
| Capnocytophaga sputigena |
| Cardiobacterium hominis |
| Corynebacterium durum |
| Corynebacterium matruchotii |
| Cryptobacterium curtum |
| Desulfobulbus sp. _R004 |
| Desulfobulbus sp. _CH031 |
| Dialister invisus |
| Dialister pneumosintes |
| Eikenella corrodens |
| Eubacterium brachy |
| Eubacterium infirmum |
| Eubacterium nodatum |
| Eubacterium saphenum |
| Eubacterium sp. BB124 |
| Eubacterium sp. BB142 |
| Eubacterium sp. DO008 |
| Eubacterium sp. IR009 |
| Eubacterium sp. strain A3MT |
| Eubacterium sulci |
| Eubacterium yurii |
| Filifactor alocis |
| Fusobacterium naviforme |
| Fusobacterium nucleatum ss. vincentii |
| Fusobacterium sp. CZ006 |
| Fusobacterium sp. R002 |
| Fusobacterium animalis |
| Fusobacterium sp._I035 |
| Fusobacterium nucleatum ss nucleatum |
| Fusobacterium nucleatum ss polymorphum |
| Fusobacterium periodonticum |
| Fusobacterium sp. BS011 |
| Gemella haemolysans |
| Gemella morbillorum |
| Granulicatella adiacens |
| Granulicatella elegans |
| Haemophilus influenzae |
| Haemophilus parainfluenzae |
| Haemophilus paraphrophilus |
| Haemophilus paraphrophaemolyticus |
| Haemophilus sp. BJ021 |
| Haemophilus segnis |
| Haemophilus sp. BJ095 |
| Kingella denitrificans |
| Kingella oralis |
| Lactobacillus casei |
| Lactobacillus rhamnosus |
| Lactobacillus zeae |
| Lactobacillus fermentum |
| Lactobacillus gasseri |
| Lactobacillus sp. HT070 |
| Lactobacillus vaginalis |
| Lautropia mirabilis |
| Lautropia sp. AP009 |
| Leptotrichia buccalis |
| Leptotrichia hofstadii |
| Leptotrichia sp. DR011 |
| Leptotrichia sp. FB074/ |
| Leptotrichia sp. BB002 |
| Leptotrichia sp. GT018 |
| Leptotrichia wadei |
| Megasphaera sp. BB166 |
| Megasphaera sp. BU057 |
| Megasphaera sp. CS025 |
| Megasphaera sp. FG014 |
| Megasphaera sp. FL008 |
| Megasphaera sp. BS044 |
| Micromonas micros |
| Micromonas sp. DA014 |
| Mycoplasma faucium |
| Mycoplasma hominis |
| Mycoplasma salivarium |
| Neisseria bacilliformis |
| Neisseria sp. AP132 |
| Neisseria polysaccharia |
| Neisseria gonorrheae |
| Neisseria meningitidis |
| Neisseria elongata |
| Neisseria sp. AP015 |
| Neisseria flavescens |
| Neisseria sicca |
| Neisseria flava |
| Neisseria macacae |
| Neisseria mucosa |
| Neisseria sp. AP060 |
| Neisseria pharyngis |
| Neisseria sp. strain B33KA |
| Olsenella genomospecies C1 |
| Peptostreptococcus micros L97 |
| Peptostreptococcus micros II VO5 |
| Peptostreptococcus sp. CK035 |
| Porphyromonas catoniae |
| Porphyromonas endodontalis Cluster: |
| Porphyromonas gingivalis |
| Porphyromonas sp. BB134 |
| Porphyromonas sp. BR037 |
| Porphyromonas sp.EP003 |
| Porphyromonas sp. CW034 |
| Porphyromonas sp.DS033 |
| Porphyromonas sp. DP023 |
| Prevotella (Bacteroides) heparinolytica |
| Prevotella buccae |
| Prevotella loeschii |
| Prevotella sp. GU027 |
| Prevotella strain B31FD |
| Prevotella sp. AO036 |
| Prevotella denticola |
| Prevotella sp. AH005 |
| Prevotella intermedia |
| Prevotella melaninogenica |
| Prevotella nigrescens |
| Prevotella oralis |
| Prevotella sp._F045 |
| Prevotella oulora |
| Prevotella pallens |
| Prevotella sp. AH125 |
| Prevotella sp. BE073 |
| Prevotella sp. BI027 |
| Prevotella sp. CY006 |
| Prevotella sp. FL019 |
| Prevotella sp. DO022 |
| Prevotella sp. DO027 |
| Prevotella sp. DO039 |
| Prevotella sp. FM005 |
| Prevotella sp. HF050 |
| Prevotella tannerae |
| Propionibacterium acnes |
| Propionibacterium sp. strain FMA5 |
| Pseudomonas aeruginosa |
| Rhodocyclus sp. strain A08KA |
| Rothia dentocariosa |
| Rothia mucilaginosa |
| Selenomonas dianae |
| Selenomonas flueggii |
| Selenomonas infelix |
| Selenomonas noxia |
| Selenomonas sp. AA024 |
| Selenomonas sp. AH132 |
| Selenomonas sp. AJ036 |
| Selenomonas sp. CI002 |
| Selenomonas sp. CS002 |
| Selenomonas sp. CS015 |
| Selenomonas sp. CS024 |
| Selenomonas sp. DD020 |
| Selenomonas sp. DM071 |
| Selenomonas sp. DS051 |
| Selenomonas sp. EW076 |
| Selenomonas sp. EW079 |
| Selenomonas sp.JS031 |
| Selenomonas sp.EW084 |
| Selenomonas sp. DS071 |
| Selenomonas sp. EZ011 |
| Selenomonas sputigena |
| Streptococcus sanguis |
| Streptococcus sp.C3 |
| Streptococcus gordonii |
| Streptococcus mitis |
| Streptococcus oralis |
| Streptococcus anginosis |
| Streptococcus intermedius |
| Streptococcus australis |
| Streptococcusinfantis |
| Streptococcus pneumoniae |
| Streptococcus sp. DN050 |
| Streptococcus sp. BE024 |
| Streptococcus sp. AA007 |
| Streptococcus sp. FN051 |
| Streptococcus constellatus |
| Streptococcus cristatus |
| Streptococcus mitis biovar 2 |
| Streptococcus mutans |
| Streptococcus parasanguinis |
| Streptococcus salivarius |
| Streptococcus sobrinus |
| Streptococcus sp. FN042 |
| Streptococcus sp. Hans new |
| Streptococcus sp. C6 |
| Streptococcus sp. C3 |
| Streptococcus sp. P4 |
| Streptococcus sp. 7A |
| Synergistes sp. _D084 |
| Synergistes sp. _W028 |
| Synergistes sp. _W090 |
| Synergistes sp. BB062 |
| Synergistes sp. BH017 |
| Tannerella forsythia |
| Tannerella sp. BU063 |
| TM7 sp. _I025 |
| TM7 sp. AH040 |
| TM7 sp. BU080 |
| TM7 sp. BE109 |
| Treponema 04:17B:maltophilum |
| Treponema 08:A:pectinovorum |
| Treponema denticola |
| Treponema lecithinolyticum |
| Treponema medium |
| Treponema socranskii (all sub-species) |
| Treponema sp. AT039 |
| Treponema vincentii |
| Veillonella dispar |
| Veillonella atypica |
| Veillonella parvula |
| Veillonella parvula |
| Veillonella sp. BU083 |
| Veillonella sp. AA050 |
| Veillonella sp. _X042 |

**Clusters in Version II HOMIM**

| Cluster Probe Name | Probe_ID | Cluster_Targets |
| --- | --- | --- |
| Actinomyces Cluster I | AB35 | All species |
| Bifidobacterium Cluster I | E49 | dentium, Parascardovia denticolens, Cryptobacterium curtum, CX010, A32ED, C1A_55 |
| Bifidobacterium Cluster II | W96 | denticolens, C5AKM003, H6-M6 and Parascardovia denticolens |
| Burkholderia Cluster I | AB64 | BRV22, mallei, cepacia, thailandensis, gladioli, glumae, xenovorans |
| Burkholderia Cluster II | AB65 | cepacia, multivorans, thailandensis, gladioli, stabilis, pyrrocinia, glumae, vietnamiensis, ubonensis |
| Campylobacter Cluster I | T87 | showae curvus rectus |
| Campylobacter Cluster II | X37 | showae curvus rectus |
| Capnocytophaga Cluster I | X24 | BM058 BU084 BR085 |
| Capnocytophaga Cluster II | AA89 | gingivalis, AH105, S3, BB167, granulosa, Strain TFI Cap08 |
| Cardiobacterium Cluster I | AB37 | valvulum, sp. A, sp. B |
| Enterococcus Cluster I | AB13 | saccharominimus, saccharolyticus, casseliflavus |
| Enterococcus Cluster II | AB43 | saccharominimus, saccharolyticus, casseliflavus, faecalis |
| Helicobacter Cluster I | AC64 | pylori, trogontum, cynogastricus |
| Lactobacillus Cluster I | W94 | rhamnosus, paracasei, casei |
| Leptotrichia Cluster I | AA45 | buccalis, IK040, GT018, GT020, C3MKM102 |
| Neisseria Cluster I | Y60 | polysaccharia gonorrhea meniningitidis |
| Neisseria Cluster II | O76 | polysaccharea, meningitidis, gonhorreae |
| Neisseria Cluster III | O45 | mucosa sicca flava macacae AP015 |
| Neisseria Cluster IV with E corrodens | Y57 | AP085, AP015, elongata, E. corrodens |
| Neisseria Cluster V | Y58 | AP067, AP060, AP085, BM052 |
| Porphyromonas Cluster I | AA46 | BR037 DP023 EP003 |
| Prevotella Cluster I | Y64 | loeschii GU027 B31FD |
| Prevotella Cluster II | Y65 | GU027 |
| Prevotella Cluster III | X13 | multiformis, AH005, denticola, FO012 |
| Prevotella Cluster IV | AB01 | DO022 FM005 |
| Prevotella Cluster V | AA50 | IK062 ID019 I |
| Prevotella Cluster VI | AA44 | pallens, nigrescens, melaninogenica, FO012 |
| Pseudomonas Cluster I | O96 | aeruginosa, pseudoalcaligenes , fluorescens, AZ002 |
| Ralstonia Cluster I | AC20 | solanacearum, pickettii, solanacearum, mannitolilytica |
| Selenomonas Cluster I | AC13 | infelix EY047 GT010 IK004 |
| Selenomonas Cluster II | AB61 | GI064 FT050 DO042 |
| Staphyococcus Cluster I | AB42 | warneri, aureus, caprae, epidermidis |
| Streptococcus Cluster I | AB98 | mitis bv2 pneumoniae peroris BW019 CH006 BM035 |
| Streptococcus Cluster II | Q59 | sanguinis salivarius Hans H6, some clones |
| Streptococcus Cluster III | Q65 | All species |
| Streptococcus Cluster IV | Q62 | anginosus, intermedius, 17 bases match sinensis, pneumoniae, parasanguis, oralis, mitis, infantis, T4-E3 |
| Streptococcus Cluster V | X09 | sobrinus, sinensis, sanguinis, salivarius, pyogenes, pneumoniae, peroris, anginosus, parasanguis |
| Synergistes Cluster I | D70 | JV006, 01, _W090, _W028, _D085, JV023, _D006, BB062 |
| Synergistes Cluster II | AC56 | BH017, _D084, JV006 |
| Treponema 5 All | AC41 | amylovorum, _U007A , BZ013, AT040, Smibert-3 D36 |
| Treponema Cluster I | AA63 | socranskii all strains, pectinovorum, parvum, putidum, MB4_G11 |
| Treponema Cluster II | AA64 | putidum, medium, denticola and clones |
| Treponema 3 All no AT004 | AC44 | _D024, _U008A, BA213, BQ032, AT013, BB003, MB@_G19, MB3_P23 |
| Treponema 3 All no BA213 | AC43 | _D024, _U008A, BQ032, AT013, BB003, MB@_G19, MB3_P23, AT003 |
| Treponema socranskii complex I | AC38 | 6:G:G47, 6:F:C44 |
| Veillonella Cluster I | D96 | dispar, BU083, AA050, BI029, _X031 |
| Veillonella Cluster II | Q67 | atypica, parvula, dispar, BU083, AA050 |
| Veillonella Cluster III | W87 | dispar, BU083, AA050 |
| Veillonella Cluster IV no atypica | AC37 | _X042, _X002, parvula, BU083, AA050, dispar, _X031 |
